# Author Correction: HuR regulates telomerase activity through TERC methylation

**DOI:** 10.1038/s41467-018-05213-5

**Published:** 2018-07-10

**Authors:** Hao Tang, Hu Wang, Xiaolei Cheng, Xiuqin Fan, Fan Yang, Mengmeng Zhang, Yanlian Chen, Yuyang Tian, Cihang Liu, Dongxing Shao, Bin Jiang, Yali Dou, Yusheng Cong, Junyue Xing, Xiaotian Zhang, Xia Yi, Zhou Songyang, Wenbin Ma, Yong Zhao, Xian Wang, Jinbiao Ma, Myriam Gorospe, Zhenyu Ju, Wengong Wang

**Affiliations:** 1 Department of Biochemistry and Molecular Biology, Beijing Key Laboratory of Protein Posttranslational Modifications and Cell Function, School of Basic Medical Sciences, Peking University Health Science Center, 38 Xueyuan Road, Beijing 100191, China; 2 Department of Physiology and Pathophysiology, School of Basic Medical Sciences, Peking University Health Science Center, 38 Xueyuan Road, Beijing 100191, China; 3 Key Laboratory of Regenerative Medicine of Ministry of Education, Institute of Aging and Regenerative Medicine, Jinan University, Guangzhou 510632, China; 4 Institute of Aging Research, Hangzhou Normal University, School of Medicine, Hangzhou 311121, China; 5 Department of Biochemistry, School of life Sciences, Fudan University, 2005 Road Songhu, Shanghai 200433, China; 6 Key Laboratory of Gene Engineering of the Ministry of Education, State Key Laboratory of Biocontrol, School of Life Sciences, Sun Yat-sen University, Guangzhou 510006, China; 7 Department of Pathology and Biological Chemistry, University of Michigan, 1301 Catherine Street, Ann Arbor, MI 48105 USA; 8 Laboratory of Genetics and Genomics, National Institute on Aging, National Institutes of Health, 251 Bayview Blvd., Baltimore, MD 21224 USA

Correction to: *Nature Communications*; 10.1038/s41467-018-04617-7; published online 07 June 2018

In the original version of this Article, the affiliation details for Fan Yang incorrectly included ‘Leibniz Institute for Age Research - Fritz Lipmann Institute, Friedrich-Schiller University of Jena, Jena, 07745, Germany’. This has now been corrected in both the PDF and HTML versions of the Article.

