# Supplementary material for: HuR regulates telomerase activity through TERC methylation
Source: Nat Commun. 2018 Jun 7;9:2213. doi: 10.1038/s41467-018-04617-7 (PMC5992219; doi:10.1038/s41467-018-04617-7)
Supplement: Supplementary file 1 — Supplementary Information [file 41467_2018_4617_MOESM1_ESM.pdf]

HuR regulates telomerase activity through *TERC* methylation

Tang et al

Supplementary Information

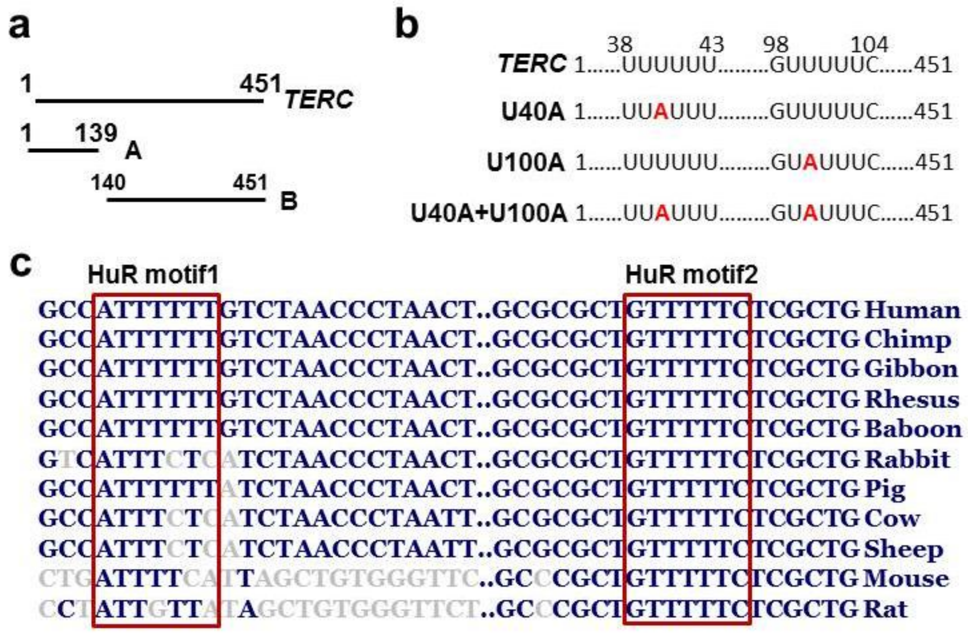

Supplementary Figure 1. ***TERC*** **HuR-binding sites are conserved in mammals.**
(a) Schematic representation of the *TERC* fragments used for RNA pulldown assays.
(b) Schematic representation depicting the *TERC* variants bearing U40A, U100A, or U40A+U100A. The point mutation sites are marked in red.
(c) The conservation of HuR recognizing motifs of *TERC* in mammals were analyzed. The UUUUUU and GUUUUUC sequences and their locations in different species are indicated by the red squares.

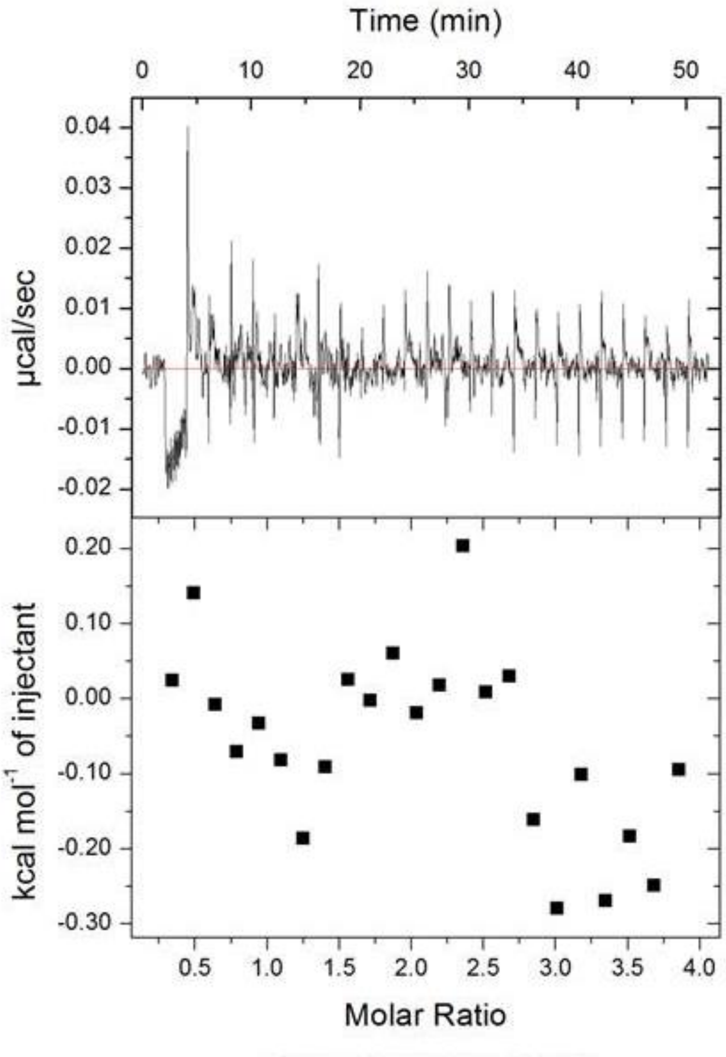

Supplementary Figure 2. Negative control for ITC assays. The RNA fragment used is CUAGACUAGGACUC.

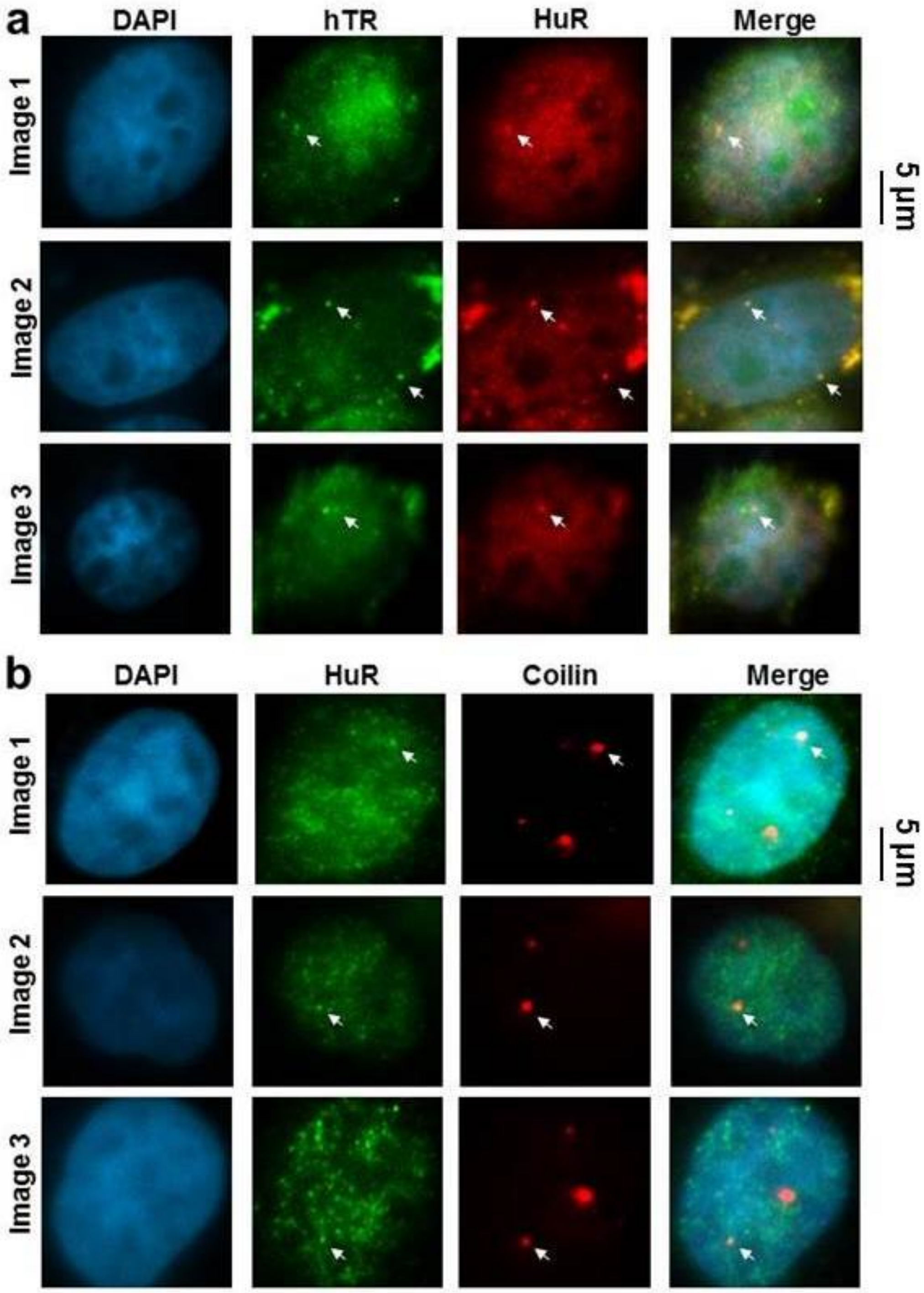

Supplementary Figure 3. Cololization of HuR with TERC and Coilin. Immunofluorescence assays were performed to test the co-localization of HuR and *TERC* **(a)**, or HuR and Coilin (a marker of Cajal bodies) **(b)**. DAPI was used to visualize nuclei. Data in (a) and (b) are 3 representative images.

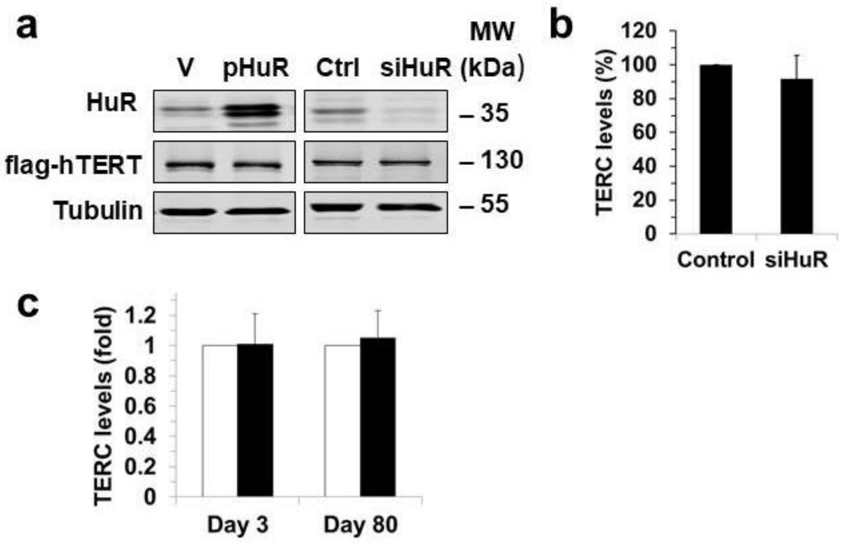

Supplementary Figure 4. HuR does not influence the expression of hTERT and *TERC*.

(a) HeLa cells were transfected with a p3×Flag-CMV 10 vector expressing flag-hTERT (containing the full-length *hTERT* mRNA). Twenty-four h later, cells were further transfected with a vector expressing flag-HuR or a HuR siRNA and cultured for an additional 48 h. The protein levels of flag-hTERT, HuR, and Tubulin were assessed by Western blot analysis. Data are representative from 3 independent experiments.
(b) RNA prepared from cells described in Supplementary Figure 4a was subjected to real-time qPCR to assess the RNA levels of *TERC*.
(c) HeLa cells were transfected with a vector expressing shNSUN2 (black) or a control shRNA (blank) for 3 or 80 days. RNA was prepared and subjected to real-time qPCR to assess the levels of *TERC*.
Data in b and c panels represent the means ±SD from 3 independent experiments.

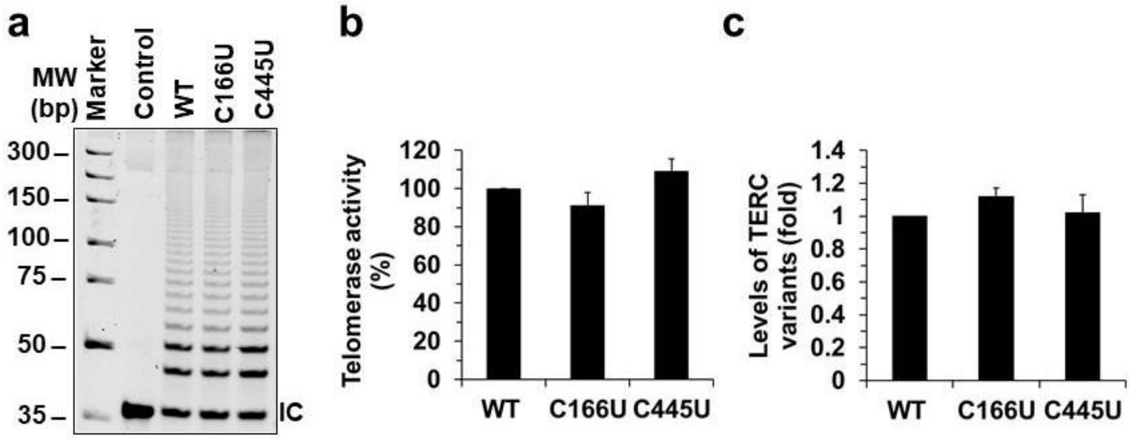

Supplementary Figure 5. Methylation at C166 and C445 in *TERC* does not affect telomerase activity. U2OS cells were transfected with a vector expressing flag-hTERT. Twenty-four h later, cells were further transfected with a vector expressing *TERC* or its variants bearing C166U or C445U and cultured for an additional 48 h. TRAP assays were performed to assess the telomerase activity **(a)**. Data were normalized against the levels of *TERC* and its variants and represented as means ± SD from 3 independent experiments **(b)**. **(c)** RNA isolated from cells described in Supplementary Figure 5a was subjected to reverse transcription (RT) followed by real-time, quantitative (q)PCR analysis to assess the levels of *TERC* and its variants bearing C166U or C445U. Data are the means ± SD from 3 independent experiments.

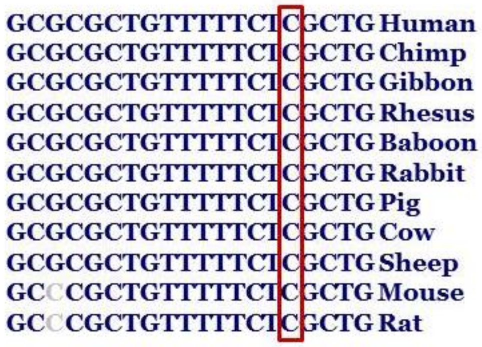

Supplementary Figure 6. Conservation of the m5C site in mammals. *TERC* sequences in different mammals were analyzed and the conserved m5C site corresponding to the C106 of *TERC* (human *TERC*) is indicated by a square.

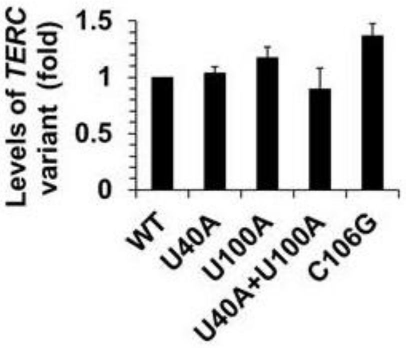

Supplementary Figure 7. Levels of *TERC* variants used for TRAP analysis shown in Figure 5c. RNA isolated from cells described in Figure 5c was subjected to RT-qPCR analysis to assess the levels of *TERC* (WT) or variants bearing U40A, U100A, U40A+U100A, or C106G. Data are the means ± SD from 3 independent experiments.

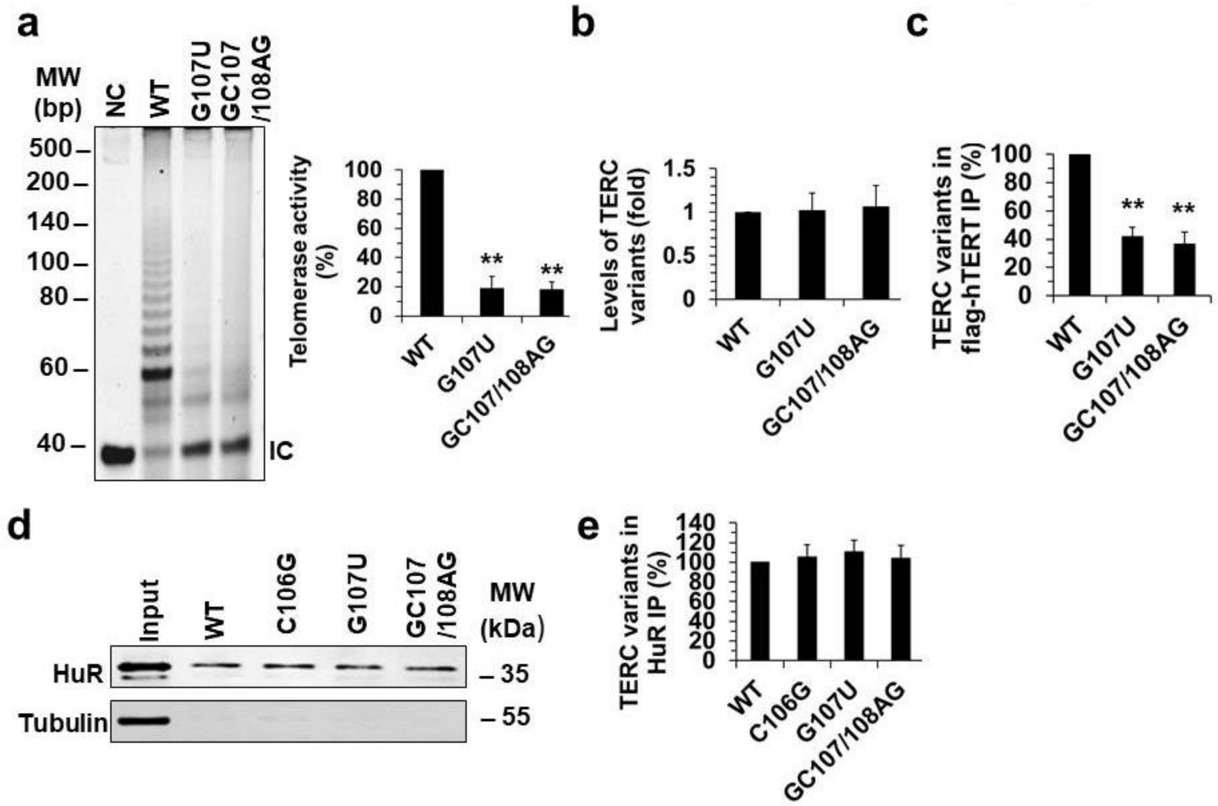

Supplementary Figure 8. C106G as well as DC-related G107U and GC107/108AG impair the association of *TERC* with hTERT, but not with HuR.

(a) U2OS cells were transfected with a vector expressing flag-hTERT. Twenty-four h later, cells were further transfected with a vector expressing *TERC*, or its variant G107U or GC107/108AG and cultured for additional 48 h. TRAP assays were performed to assess the telomerase activity (*left*). Data were normalized against the levels of *TERC* or its variants and represented as means ± SD from 3 independent experiments; significance was analyzed by Students' *t* test (*right*) (**, p < 0.01).

(b) RNA isolated from cells described in Supplementary Figure 8a was used for RT-qPCR analysis to assess the levels of *TERC* or variants bearing G107U or GC107/108AG. Data are the means ± SD from 3 independent experiments.

(c) Cells described in Supplementary Figure 8a were used for UV crosslinking followed by RNP IP assays by using an anti-flag antibody. RNA isolated from the IP materials was subjected to RT-qPCR analysis to assess the levels of *TERC* and its variants in IP against their total levels. Data are the means ± SD from 3 independent experiments; significance was analyzed by Student's *t* test (**, p < 0.01).

(d) RNA pulldown assays were performed by using HeLa cell lysates and *in vitro*-transcribed *TERC* or its variants (C106G, G107U or GC107/108AG). The presence of HuR and Tubulin in the pulldown materials was assessed by Western blot analysis. Data are representative from 3 independent experiments.

(e) U2OS cells were transfected with a vector expressing *TERC* or its variants. Forty-eight h later, lysates were used for UV crosslinking RNA IP assays by using an anti-HuR antibody. The presence of *TERC* in the IP materials relative to total levels was assessed by using RT-qPCR analysis. Data are the means ± SD from 3 independent experiments.

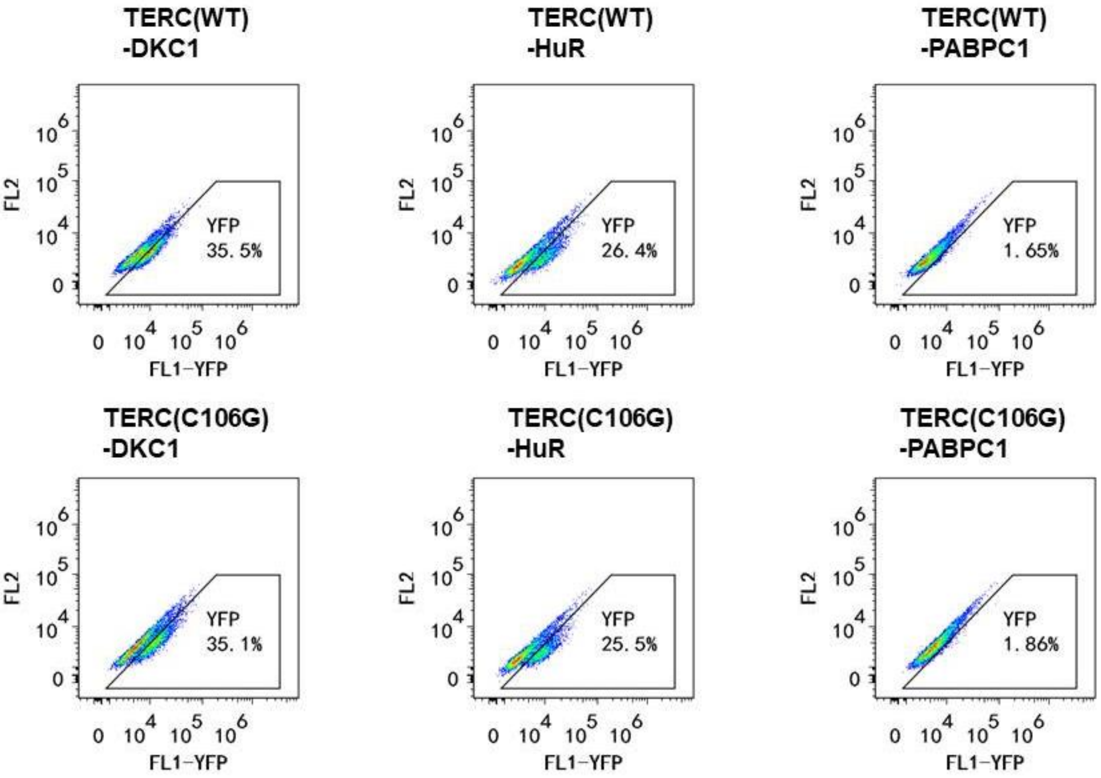

Supplementary Figure 9. C106 methylation does not influence the association of HuR with *TERC*. The TriFC system was used for detecting the interactions between HuR and *TERC* and its variant C106G in HTC75 cells by flow cytometry. DKC1 and PABPC1 served as positive and negative controls, respectively.

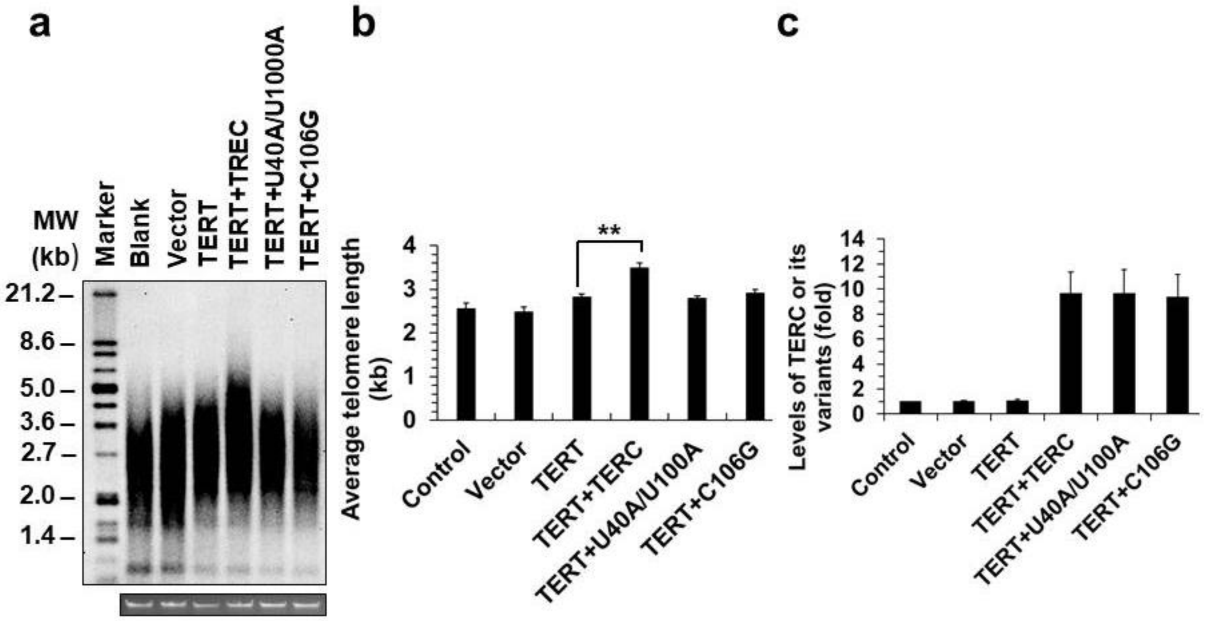

Supplementary Figure 10. Mutation of the *TERC* HuR binding motifs or methylation site impairs the effect of *TERC* in maintaining telomerase activity. **(a, b)** HeLa cells were stably transfected with a vector expressing flag-TERT (TERT), or co-transfected with a vector expressing flag-hTERT together with a vector expressing *TERC* (TERT+TERC) or variant bearing U40A+U100A (TERT+U40A/U100A) or C106G (TERT+C106G). Twenty-one days later, Southern blot analysis was performed to assess telomere length (a). Data are the means ± SD from 3 independent experiments; significance is analyzed by using Student's *t* test (**, $p < 0.01$) (b). **(c)** RNA isolated from cells described in Supplementary Figure 10a was used for real-time qPCR analysis to assess the levels of *TERC* or its variants. Data are the means ± SD from 3 independent experiments.

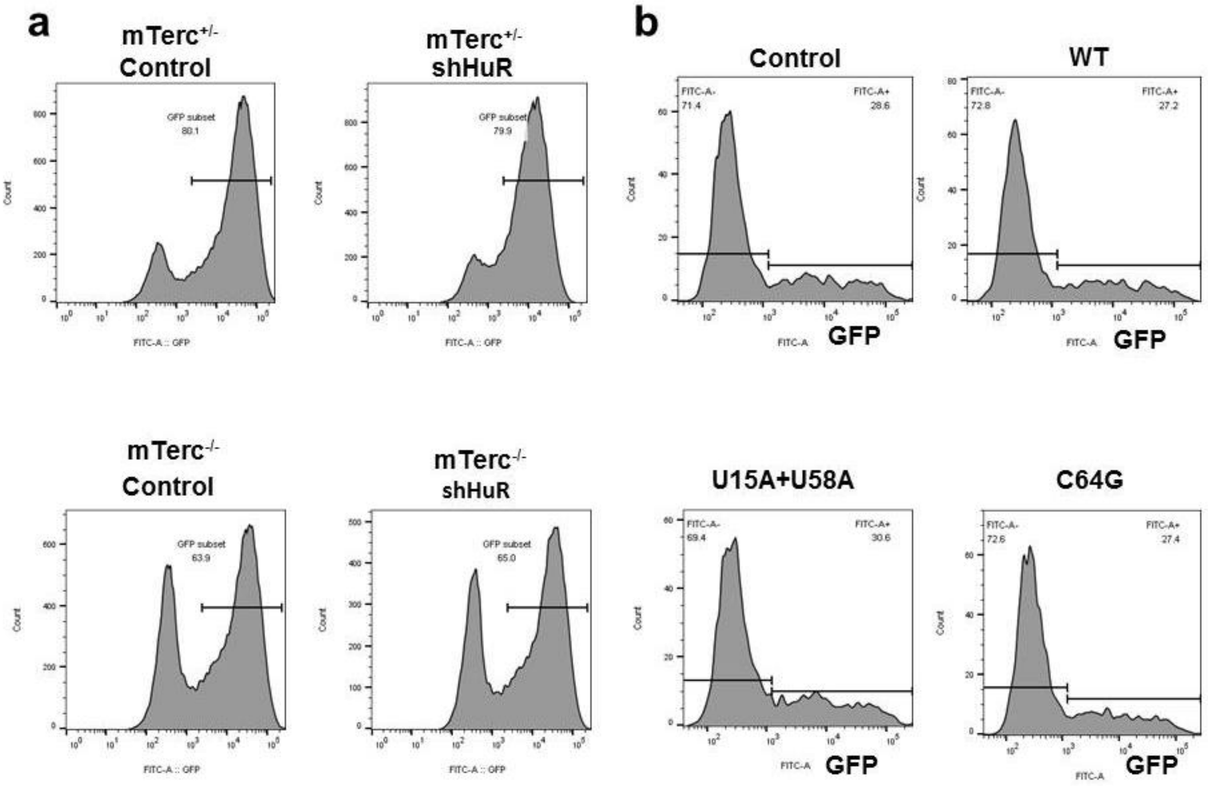

Supplementary Figure 11. The transduction efficiency in LSK cells before transplantation.

(a) Terc$^{+/-}$ or mTerc$^{-/-}$ LSK cells were infected with viruses expressing either HuR shRNA (shHuR) or a control shRNA (Control). Transduction efficiency was monitored by FACS analysis.

(b) mTerc$^{-/-}$ LSK cells were infected with a control virus (Control) or a virus expressing *mTERC* (WT), or its variants bearing U15A+U58A (U15A+U58A) or C64G (C64G). Transduction efficiency was monitored by using FACS analysis.

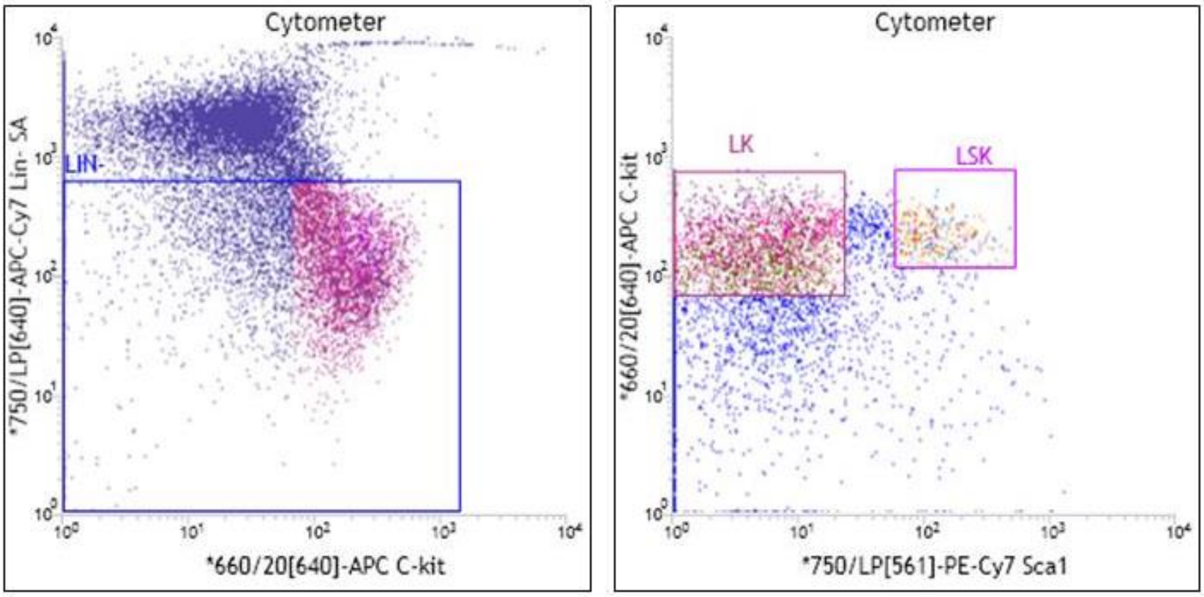

Supplementary Figure 12. Representative FACS plot for the sorting of LSK cells.
LSK cells (lineage negative, cKit and Sca-1 double positive) cells were sorted **from the MACS enriched cKit positive BM cells** for LV transduction.

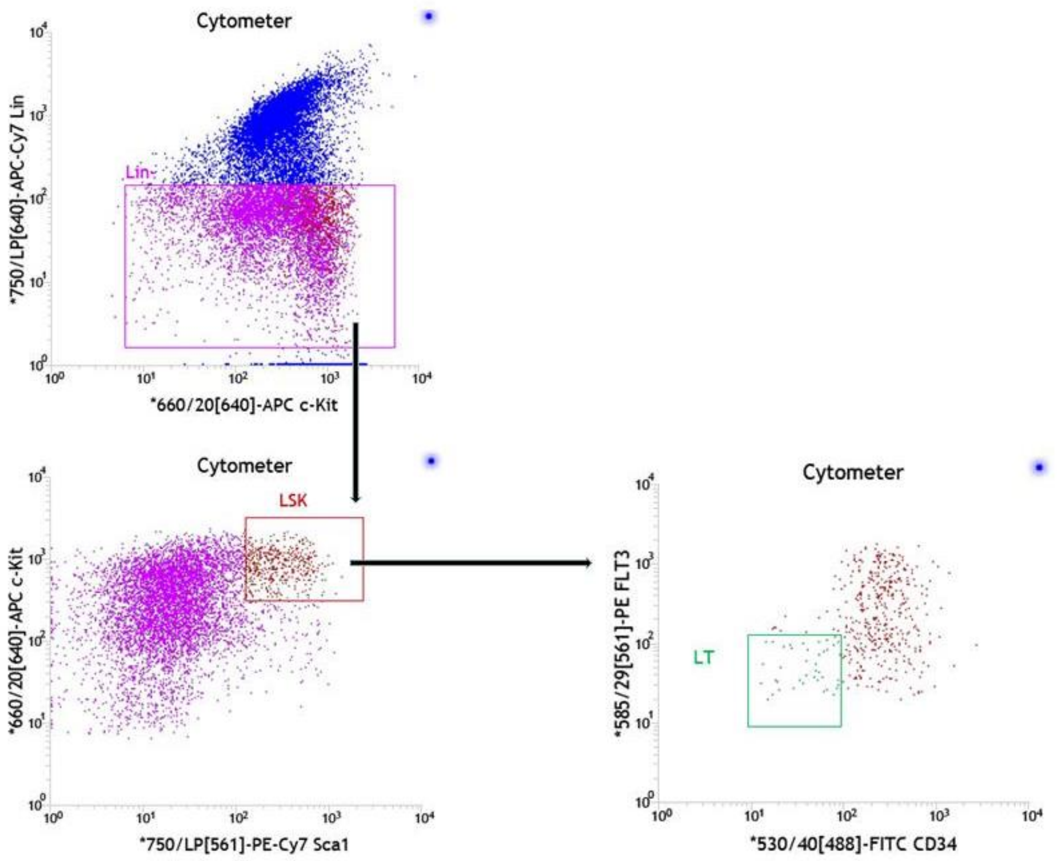

Supplementary Figure 13. Representative FACS plot for the sorting of Long term HSCs (LT-HSC). LT-HSC (LSK, Flt3 positive and CD34 low/negative cells) were sorted for telomere length analysis.

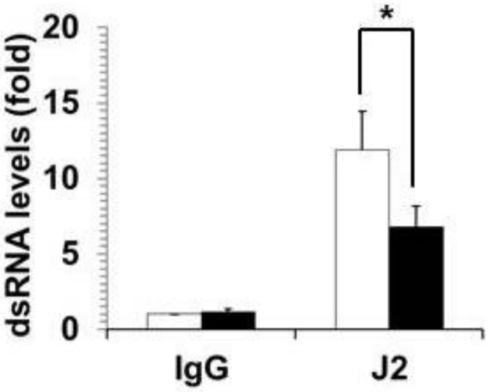

Supplementary Figure 14. HuR influences the formation of the double strand RNA (dsRNA) of *TERC.* HeLa cells were transfected with a HuR siRNA (black) or a control siRNA (blank). Forty-eight h later, cell lysates were collected by using J2 lysis buffer and subjected to dsRNA pull-down assays by using an anti-J2 antibody. The levels of dsRNA of *TERC* in the pull-down materials were analyzed by using real-time qPCR. Data are the means ± SD from 3 independent experiments; significance was analyzed by Student's *t* test (*, $p < 0.05$).

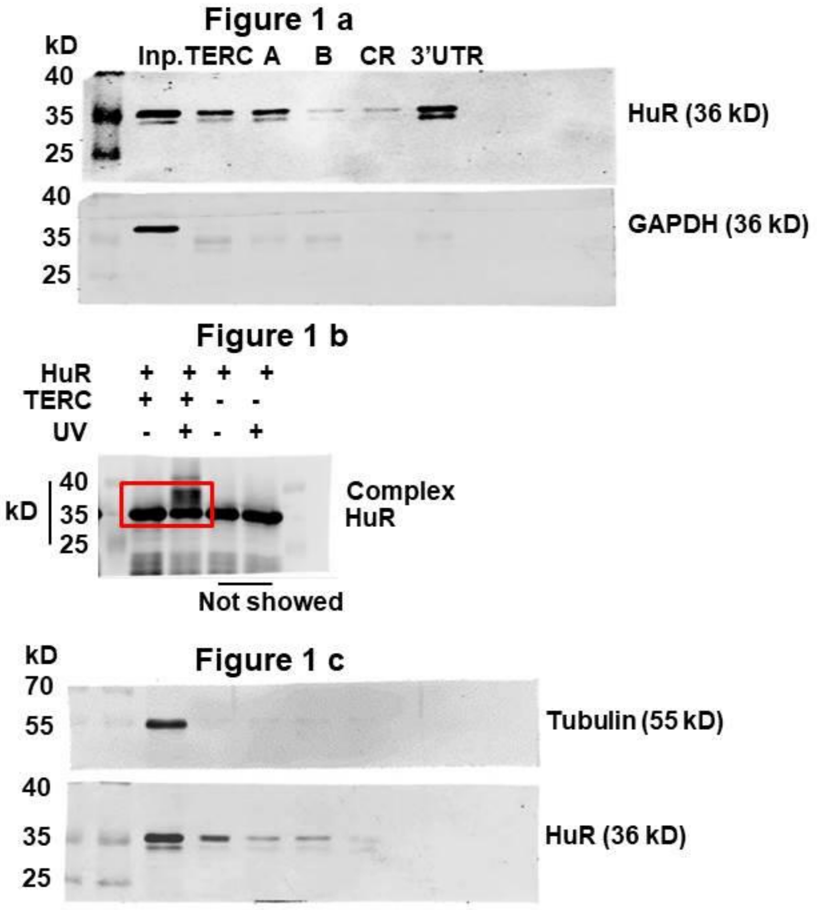

Supplementary Figure 15. Uncropped images of Western blots shown in panels a, b, and c of Figure 1 with marker molecular weight indicated. Blots presented in Figure 1b were squared.

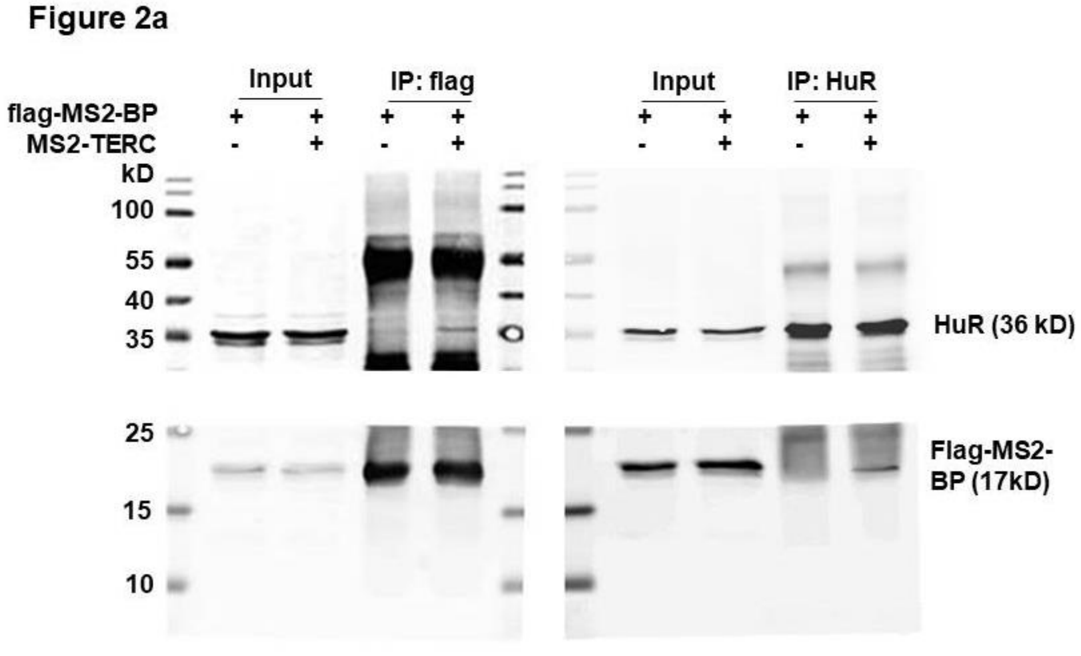

Supplementary Figure 16. Uncropped blots of Western blot analysis shown in Figure 2a with marker molecular weight indicated.

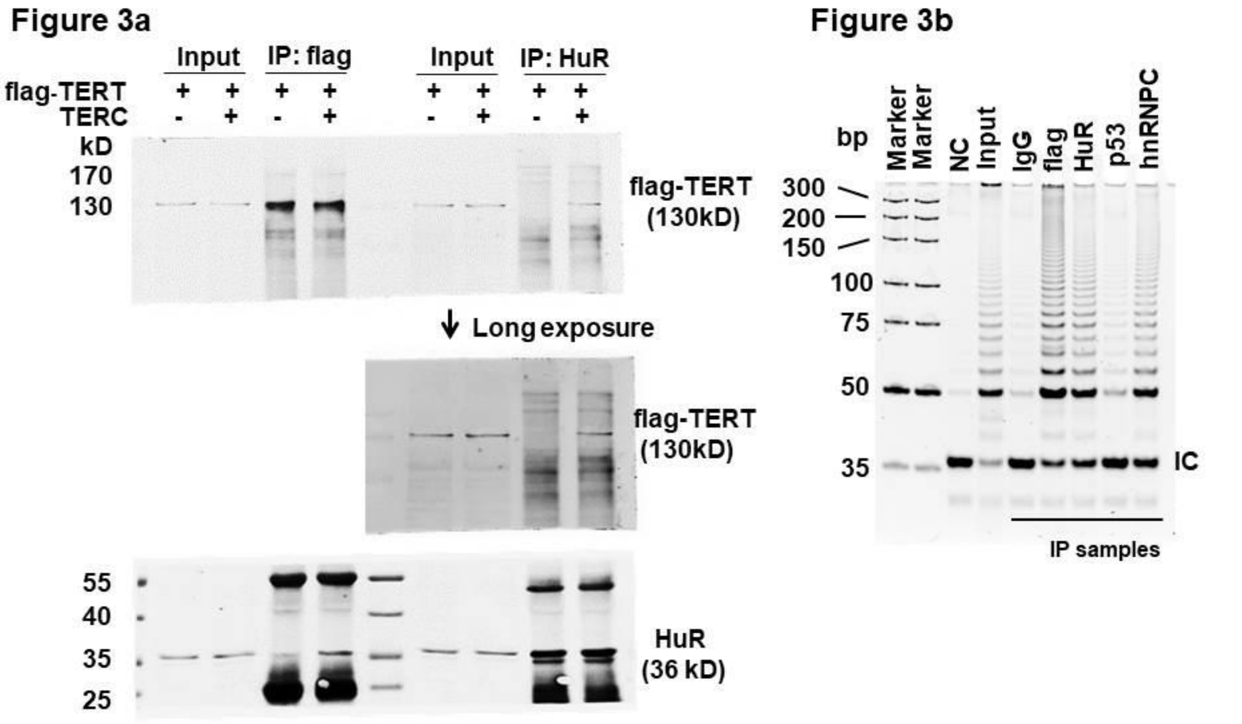

Supplementary Figure 17. Uncropped blots of Western blot analysis shown in Figure 3a and TRAP image shown in Figure 3b with marker molecular weight indicated.

Figure 4a

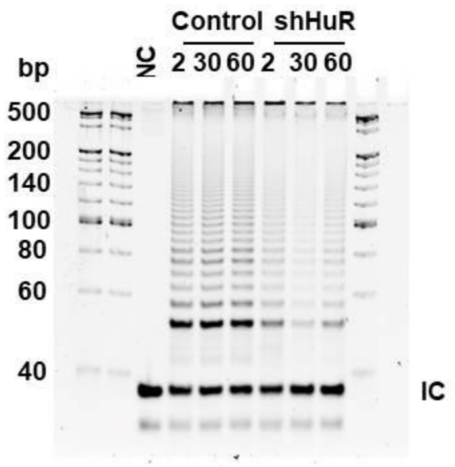

Figure 4b

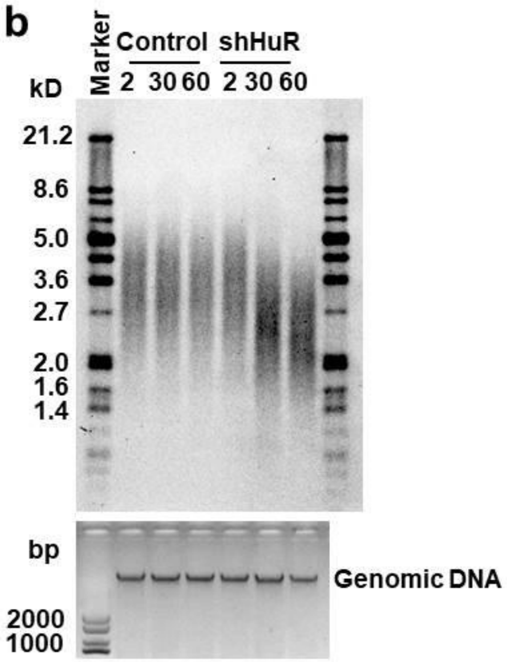

Supplementary Figure 18. Uncropped blots shown in Figure 4a (TRAP assays) and Figure 4b (Southern blot analysis) with molecular wright indicated. The loading control of the Southern blot analysis (Genomic DNA) was also included.

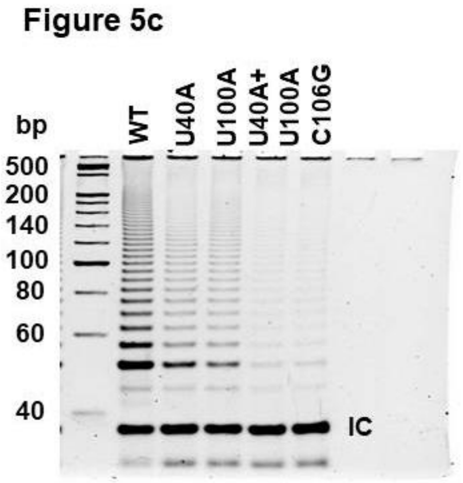

Supplementary Figure 19. Uncropped image of TRAP assays shown in Figure 5c with molecular wright indicated.

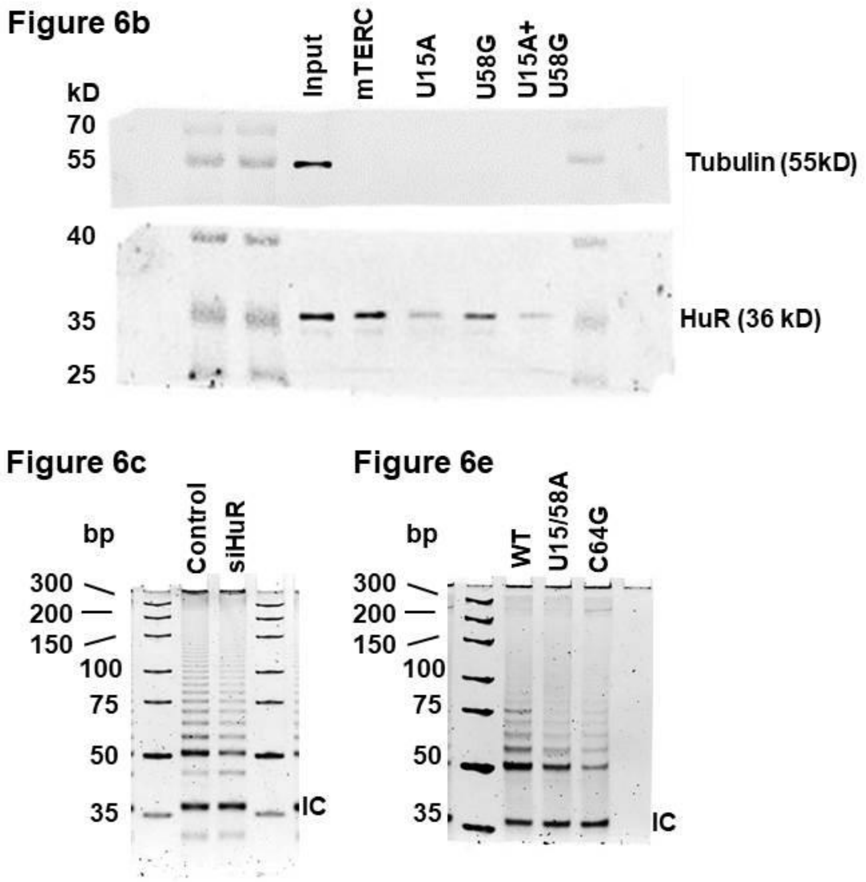

Supplementary Figure 20. Uncropped blots of Western blot analysis shown in Figure 6b and TRAP images shown in Figure 6c and 6e with marker molecular weight indicated.

Supplementary Figure 4a

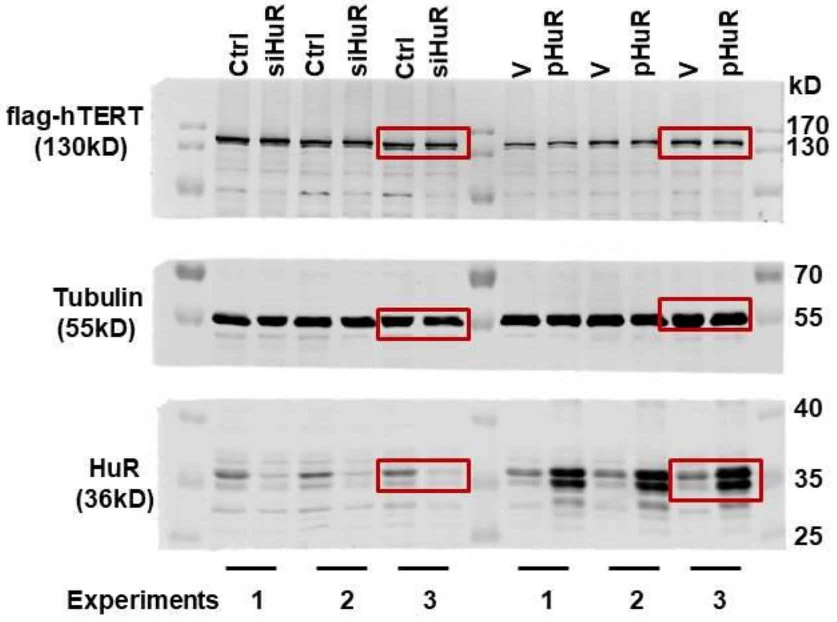

Supplementary Figure 21. Uncropped blots of Western blot analysis shown in Supplementary Figure 4a with marker molecular weight indicated. Blots presented in Supplementary Figure 4a were squared.

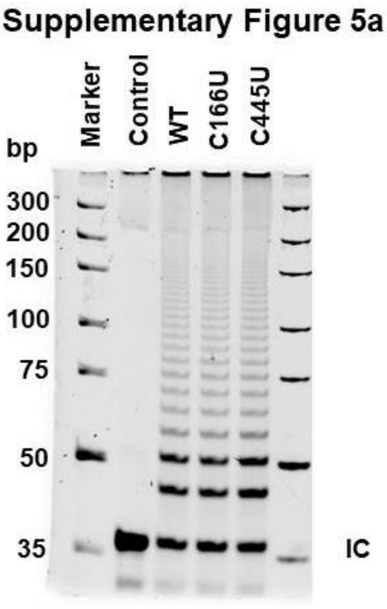

Supplementary Figure 22. Uncropped image of TRAP assays shown in Supplementary Figure 5a with marker molecular weight indicated.

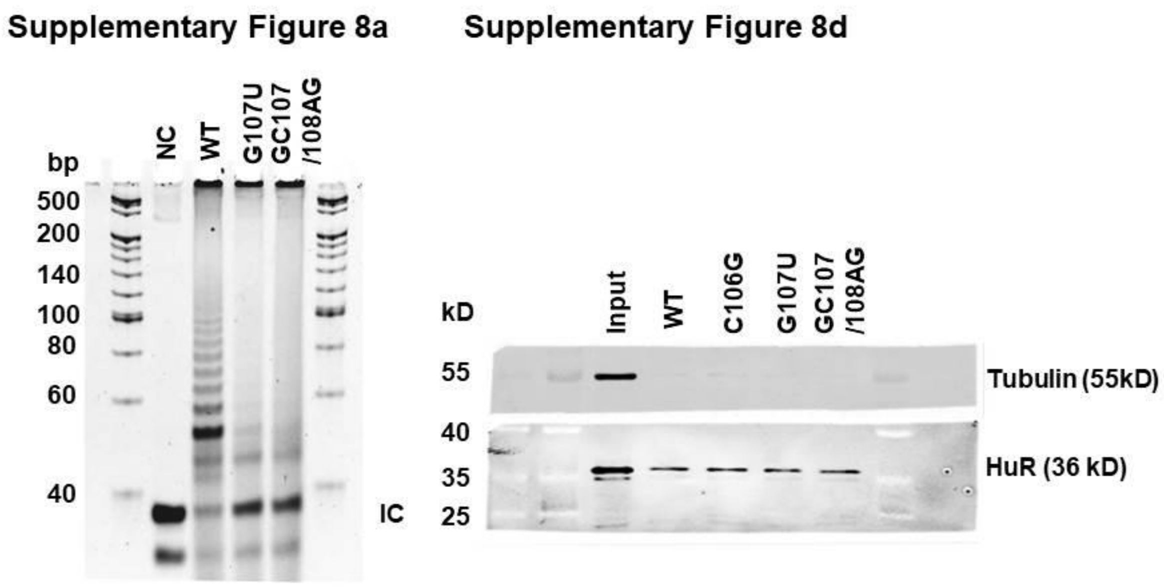

Supplementary Figure 23. Uncropped image of TRAP assays and blots of Western blots shown in Supplementary Figure 8a and 8b, respectively. The molecular weight of markers was indicated.

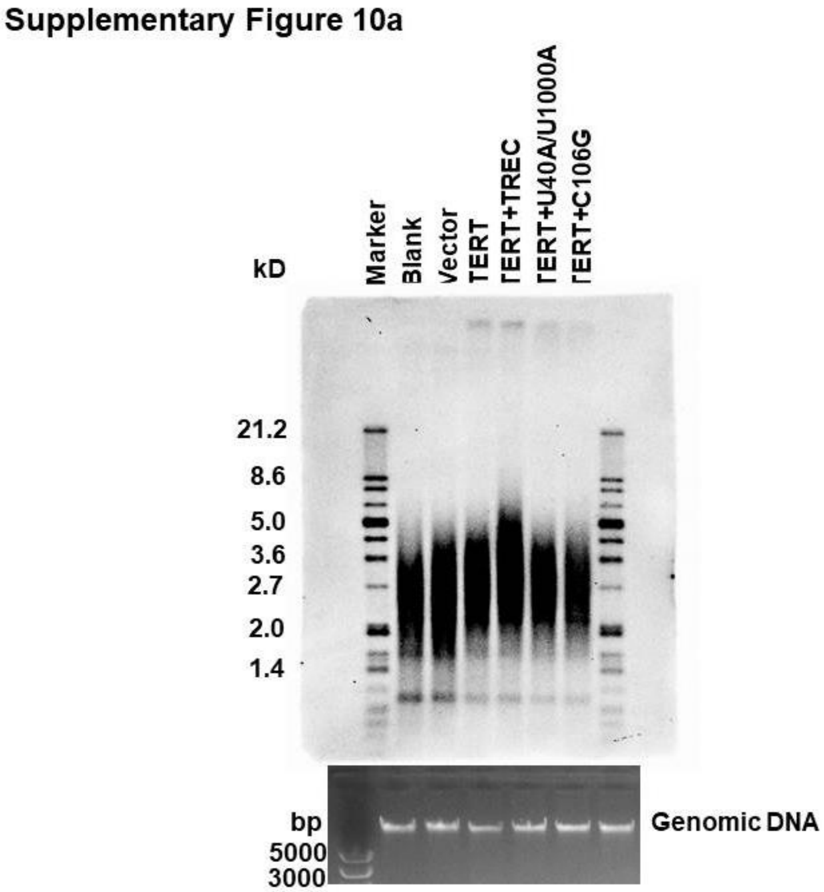

Supplementary Figure 24. *Upper*, uncropped blot of Southern blot analysis shown in Supplementary Figure 10a with molecular weight of the markers indicated. *Bottom*, the loading control (Genomic DNA) was included.
